# A simple mathematical model for evaluation of non-fragmentation property of injectable calcium-phosphate cement

**DOI:** 10.1038/s41598-025-06039-0

**Published:** 2025-07-01

**Authors:** Yu Ichida, Rika Yamada, Shiori Kato, Yuki Kamaya, Minami Kosuge, Mamoru Aizawa, Takashi Okuda Sakamoto, Shigetoshi Yazaki

**Affiliations:** 1 Department of Mathematical Sciences, School of Science, Kwansei Gakuin University, Gakuen Uegahara 1, Sanda, 669—1330 Hyogo Japan; 2 Applied Chemistry Program, Graduate School of Science and Technology, Meiji University, 1-1-1 Higashimita, Tama-ku, Kawasaki, 214—8571 Japan; 3 Meiji University International Institute for Materials with Life Functions, 1-1-1 Higashimita, Tama-ku, Kawasaki, 214—8571 Japan; 4 Department of Mathematics, School of Science and Technology, Meiji University, 1-1-1 Higashimita, Tama-ku, Kawasaki, 214—8571 Japan

**Keywords:** Paste-like artificial bone, Inositol phosphate, Non-fragmentation property, Allen-Cahn type equation with a time-dependent diffusion coefficient., Chemistry, Materials science, Mathematics and computing

## Abstract

In this study, the mechanism of fragmentation including void formation in an injectable calcium-phosphate cement of paste-like artificial bone grafting material, is clarified from the point of view of mathematical modeling. Instead of modeling from physical laws or chemical reactions, phenomenological modeling is used, and three experiments are performed to build the model. We then successfully use the results in terms of the equations and necessary assumptions to construct a simple mathematical model. Our proposed model is based on the mathematically well-known Allen-Cahn type equation. The state of the paste is used as an unknown function. After scaling according to the appropriate nondimensionalization, numerical simulations are performed. The numerical results represent the setting behavior of the paste, which is difficult to observe experimentally. The integration of mathematical and experimental results provides deep insight into the mechanism of non-fragmentation property of an injectable calcium-phosphate cement.

## Introduction

Developing treatments that are as easy as possible for patients with bone disease is one of the most important issues in orthopedics. The standard treatment for bone disease (for instance, osteoporosis) is the transplantation of one’s own bone. This method has at least two problems. First problem is the limiting the amount of bone needed for grafting. The second problem is a heavy burden on the patient.

That is, the possibility of re-operation due to another fracture after transplantation places.

An alternative to grafting of one’s own bone is the use of bioceramics to reduce the burden on the patient. For example, Hydroxyapatite [Ca_10_(PO_4_)_6_(OH)_2_; HAp] and β-tricalcium phosphate [β-Ca_3_(PO_4_)_2_; β-TCP] are known. As an application of these materials, calcium-phosphate cement (CPC) that can be injected into the bone defects has been recently developed. The CPC is a paste-like artificial bone grafting material; actually, the paste is injected into the bone defect using a syringe, etc., and hardened on site. Therefore, it is known as an artificial bone that can realize minimally invasive treatment^1^. In clinical applications of the CPC, multiple material properties (e.g., paste injectability, initial setting time, mechanical strengths, and inhibition of fragmentation *in vivo* after setting) must be achieved simultaneously. In addition, direct bonding with the host bone (bioactivity)^2^, bioresorbability^3^, and anti-bacterial properties^4^ are required depending on the application situation.

Among the above-mentioned properties, this paper focuses on fragmentation property. According to our previous report^5^, fragmentation is defined as follows: (I) the CPC does not become a lump after setting, and (II) cracks and voids are generated in the CPC during setting. Naturally, it reduces the biomechanical strength of CPCs. In addition to mechanical issues, breakage can lead to inflammation and the need for reoperation. The term for the counterpart of fragmentation property will be referred to as non-fragmentation property in this paper. It is necessary not only to develop a paste that exhibits non-fragmentation properties even after setting, but also to establish a non-destructive evaluation method for it.

Aizawa and his group^5^ previously developed a novel CPC using inositol phosphate (hereinafter to as IP6) as a chelating agent. The results of non-destruction observation using X-ray CT are obtained regarding the non-fragmentation property with and without IP6. A sample is injected into a cylindrical shape in the laboratory and the paste is set. We observe that it shows non-fragmentation property when IP6 is present, and fragmentation property in the case of water (without IP6). Why is it the non-fragmentation property in IP6? What is the mechanism? These are not solved in biomaterials. We aim to resolve these questions from the perspective of mathematical modeling.

The basis for the construction of mathematical models are physical laws and chemical reactions. However, it is difficult to express in equations the explicit chemical reactions in a series of experiments in which the paste is properly prepared, injected into a cylindrical shape, and set. Therefore, our models are built empirically using known mathematical equations. We then conduct experiments to build the mathematical model and explain the parts of the equation and the whole by skillfully considering the results. We assume that the paste is considered as a particle and based on the reaction-diffusion equation in spatial two-dimensions, in particular the Allen-Cahn type equation. The assumption of a two-dimensional space means that no matter where you cut, it is uniform. The Allen-Cahn equation is one of the representative reaction-diffusion equations and is considered one of the equations describing phase separation (see^6^ and references therein). According to^7^, it has been applied to various problems, for example, phase transition, image analysis and motion by mean curvature.

In the construction of a mathematical model, the question arises as to how to determine the diffusion and reaction terms given the two-dimensional space and the Allen-Cahn type equation. In this paper, we conduct three experiments to build a mathematical model and argue for the validity of our model construction. The first experiment is a coloring experiment to observe particle dispersion. We investigated coloration with and without IP6 using materials that bind to specific substances to produce green and red coloration, respectively. The first experiment asserts that the presence or absence of IP6 plays a role in non-fragmentation property even when there is no soaking process, simplifying the experiment in^5^ in which cylindrical shapes injected with paste were soaked in water or blood. According to this experiment, these show that the ease of cement paste spreading with and without IP6 is related to non-fragmentation. The second experiment is the experiment to use the Vicat needle. Although the time to set has been measured so far, quantitative measurement of setting, not time, is used to understand the setting behavior. This experiment allows the setting to be viewed as diffusion and included in the equation as a time-dependent diffusion coefficient. The third experiment is the experiment to observe the setting behavior for the Hele-Shaw cell. To study the setting behavior in two dimensions, the paste is injected onto the plate and crushed from above. Through this experiment, we obtain that the difference in the spreading of the paste is markedly different with the concentration of IP6 as an axis. These experiments are for mathematical modeling and have never been done before. In the view of the field of biomaterial, they gain new insights into the setting behavior.

These three experiments show that the concentration of IP6 is key and plays a significant role in the setting and the evaluation of the non-fragmentation property. Therefore, we consider a two-dimensional spatial domain and propose the Allen-Cahn type equation with time-dependent diffusion coefficients with the unknown function as an order parameter in our mathematical model. The model accounts for the concentration dependence of IP6 in the diffusion coefficient and reaction term, and successfully incorporates the three experiments described above. Then, by introducing a scaling transformation to reduce the number of IP6 concentration-dependent parts, and by numerical simulation. The non-fragmentation property can be shown when IP6 is sufficiently present, and the process of paste setting behavior, which is difficult to observe experimentally, can be obtained.

Our model is based on the Allen-Cahn type equation, a well-known mathematical model, but captures the setting behavior in a simple and essential form. It provides one insight into the unresolved setting behavior of paste. It would be the first step in giving the essential and fundamental mechanism of the non-fragmentation property with and without IP6 in more general situations, such as experimental systems and animal experiments in^5^. In addition, it mathematically gives a new application of the Allen-Cahn type equation.

Even if it is possible to construct a mathematical model that considers all the fragmentation processes of an injectable calcium-phosphate cement of paste-like artificial bone grafting, it is meaningless since it is impossible to analyze the model. Therefore, this manuscript attempts a qualitative approach by introducing order parameters from a phenomenological point of view. Our approach is a standard in mathematical modeling theory, and the series of experiments are modeled by considering the essence of the process as a change in the concentration of IP6. To the best of the authors’ knowledge, no studies on mathematical modeling of the phenomena discussed in this phenomenon exist. This study is innovative since it conducts three experiments to verify the validity of a mathematical model constructed using a phenomenological approach and clarifies how the model indicates the difference in the paste’s setting behavior with and without IP6. In addition, the governing equations constructed are those that move from partial differential equations to ordinary differential equations. From the viewpoint of mathematical modeling, our constructing equation shows many new mathematical problems in terms of numerical and analytical treatment (e.g., adequacy).

This paper is organized as follows. The next section presents the three key experiments concluded to build the mathematical model and their results. Section 3 describes our proposed model in detail. Section 4 integrates the mathematical model and its numerical results with conventional biomaterials knowledge to provide some insights into the mechanism of the non-fragmentation property with and without IP6.

## Experiments for constructing mathematical model

### Coloring experiment to observe particle dispersion

Firstly, two types of starting powders for cement fabrication, that is, β-TCP powder with and without IP6, were prepared according to a previous report^5^. Briefly, IP6/β-TCP powder was obtained by adding β-TCP powder and IP6 solution with the concentration of 3000 ppm to the zirconia pot of a ball mill, and then by ball-milling under wet conditions using zirconia beads with 2 mm diameter. Water/β-TCP powder was obtained by the same operation in pure water, but not IP6 solution.

**Fig. 1:**
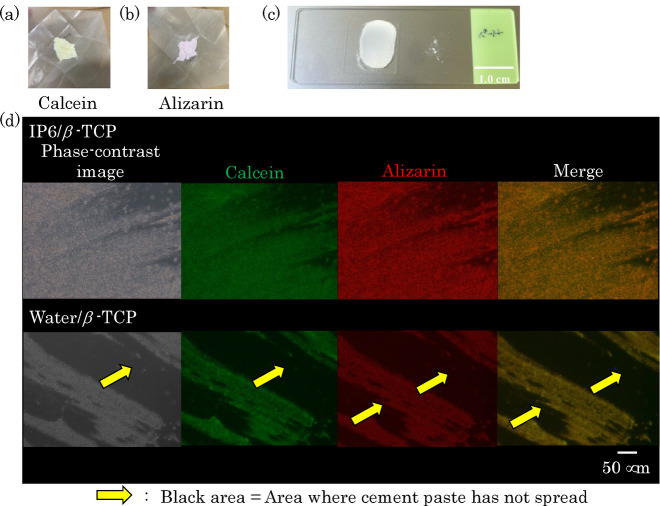
Coloring experiment to observe particle dispersion: IP6/β-TCP powder loaded with (**a**) calcein and (**b**) arizarin, (**c**) appearance of paste extended on a glass slide, and (**d**) phase contrast and fluorescence micrographs of the stretched paste in (**c**) (calcein- and alizarin-loaded powders are stained green and red, respectively).

Calcein and alizarin red solutions were prepared at 50 ppm each using pure water. Firstly, 3 g of IP6/β-TCP and water/β-TCP powders were added to 15 cm³ of the pure water, respectively, and then stirred in a vortex mixer for 1 min. The powders were then centrifuged, the supernates were removed, and the powders were lyophilized. The lyophilized powder was then sieved through a 100-mesh screen to obtain the dye powder. Figure 1a, b shows the overviews of the resulting powders stained with calcein and alizarin, respectively. Note that calcein is specifically adsorbed on calcium and shows green color under fluorescence microscopy. Alizarin red also adsorbs specifically to calcium, and shows a red color. These calcein- and alizarin red-stained powders were mixed with a mortar and pestle to a mass ratio of 1:1, and then mixed with mixing liquid for 2 min to a powder-liquid ratio (P/L ratio) of 1/0.9 [w/w]. The present mixing liquid for cement fabrications was prepared by dissolving disodium hydrogen phosphate (2.5 g), sodium alginate (1.0 g), and citric acid (1.5 g) into pure water (95 g), and adjusting the pH to 7.0 with a sodium hydroxide solution. The resulting paste was extended onto a glass slide (Fig. 1(c)) and observed using a fluorescence microscope.

The results of the fluorescence microscopic observation are shown in Fig. 1d. In the phase-contrast images, black areas were observed in the Water/β-TCP sample, whereas the IP6/β-TCP sample showed relatively uniform spreading of the paste with almost no black areas. The black areas indicate the absence of stained cement particles. The calcein and alizarin red stained images also showed relatively homogeneous spreading of green and red in the IP6/β-TCP sample; the merge image is a superimposition of the calcein and alizarin red images. These observations indicate that in the presence of IP6, (i) the cement paste spreads relatively homogeneously and (ii) the β-TCP particles in the hardened cement are uniformly mixed. In fact, to our knowledge, Water/β-TCP causes fragmentation in some cases, while IP6/β-TCP does not. The uniform spreading of the paste is considered to homogenize the density of particles in the final hardened product, thus inhibiting the formation of microcracks that cause fragmentation.

### Experiment to determine setting time using the Vicat needle

**Fig. 2:**
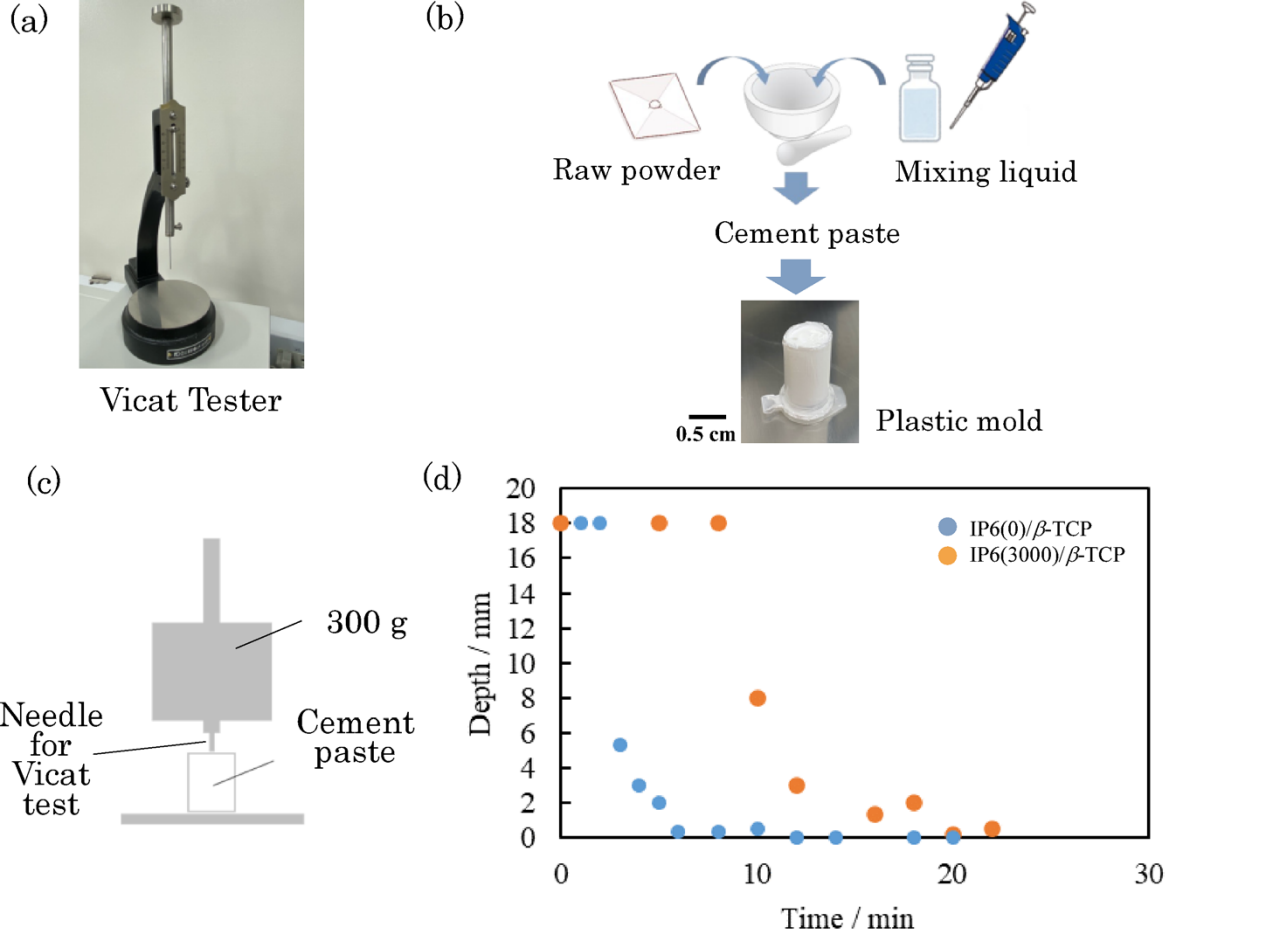
Experiment to use the Vicat needle: (**a**) appearance of Vicat needle tester, (**b**) flow of the Vicat needle test, (**c**) schematic diagram of the depth measurement, and (**d**) depth of penetration of Vicat needle into IP6/β-TCP and Water/β-TCP pastes filled in plastic molds over time.

In this section, the setting time of cement paste was examined by a Vicat needle test on the basis of JIS T0330-4. The appearance of the Vicat needle tester used is shown in Fig. 2a. Firstly, two types of cement pastes with and without IP6 were prepared as in Sect. 2.1. These pastes were filled into a designated plastic mold (Fig. 2b). The plastic molds filled with the pastes were placed in an incubator set at 37 °C, and depth measurements were taken with a Vicat needle (mass 300 g) after each designated time interval. As shown in the diagram modeled in Fig. 2c, the depth measurement was performed by setting the Vicat needle on the uppermost surface of the plastic one and measuring how much the needle penetrated into the paste under its own weight. We defined the setting time as the time when the needle no longer penetrated into the paste (0 mm depth).

Figure 2(d) shows the change over time of the penetration depth of a Vicat needle into IP6/β-TCP and Water/β-TCP pastes filled into a plastic mold. The depth of penetration of the Vicat needle was large immediately after the start of measurement for both pastes, however, after a certain amount of time, the penetration of the needle suddenly became difficult, and finally the needle did not penetrate and the pastes reached the final setting. The final setting of IP6/β-TCP took longer than that of Water/β-TCP. The surface potentials of the particles constituting these two IP6/β-TCP and Water/β-TCP pastes were measured by Doppler laser scattering (ELSZ-2, Otsuka Electronics, Japan) and were − 17.3 ± 0.1 mV for IP6/β-TCP and − 12.1 ± 0.7 mV for Water/β-TCP. Errors are shown as standard deviations. This experiment was conducted at least three times. Figure 2d shows the representative results. The IP6/β-TCP had a more negative charge than Water/β-TCP. This can be explained by the fact that IP6 has 12 OH groups, which means that when IP6 is chemically modified into β-TCP, it has a stronger negative charge than β-TCP without IP6. IP6/β-TCP has a higher negative charge than Water/β-TCP since IP6 is adsorbed on TCP particles. Considering colloidal stability, this can be said to increase colloidal stability because the negatively charged particles are more repulsive to each other. The result is attributed to the phenomenon of well spreading of the paste, as shown in Fig. 1d.

During the setting process of the paste, aggregation and rearrangement among individual particles are important factors. Considering the electrostatic repulsion among particles, IP6/β-TCP particles are more negatively charged than Water/β-TCP particles, resulting in the repulsion among particles, which may lead to the (i) aggregation and subsequent (ii) rearrangement of particles and (iii) gradual interaction between IP6 and calcium on the β-TCP particle surface. On the other hand, as the negative charge among the particles of Water/β-TCP is not relatively strong, the aggregation among the particles is considered to have occurred somewhat quickly, leading to the setting. Although such rapid setting is one of the advantages for the CPCs, it may be one of the factors causing microcracks to form because it makes the density distribution of particles in the final cement non-uniform.

### Experiment to observe setting behavior for the Hele-Shaw cell

Through the two experiments in Sect. 2.1 and 2.2, it was found that the presence or absence of IP6 contributed to the formation of microcracks. Therefore, in this section, using pastes with varying amounts of IP6 loading on β-TCP, we examined the paste spreading and crack formation by Hele-Shaw cell, and observed the microstructure of the setting body using a scanning electron microscope (JSM-6390LA, JEOL, Japan). In a similar study, Aizawa et al.^8^ prepared IP6/β-TCP cements by varying the concentration of IP6 surface modification from 0 to 10,000 ppm. They then determined the material properties of these cements. The study revealed that the material properties were optimal at an IP6 concentration of 3,000 ppm (e.g., compressive strength and anti-washout ability). Implanting the paste into a porcine tibia demonstrated its excellent bone formation and bioresorption properties. Based on these findings, the experiment for the Here-Shaw cell conducted experiments using pastes with varying IP6 concentrations ranging from 0 to 10,000 ppm.

Firstly, the starting powders for cement fabrications were prepared in the same manner as described in Sect. 2.1. In addition to IP6 concentrations of 0 (pure water) and 3000 ppm, the IP6/β-TCP powders of 1000, 5000, and 10,000 ppm were also prepared. Hereafter, for example, the paste prepared with an IP6 concentration of 3000 ppm will be referred to as “IP6(3000)/β-TCP”. The mixing solution for cement preparation was the same as in Sect. 2.1 and 2.2.

**Fig. 3:**
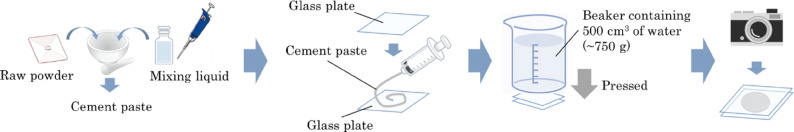
Schematic diagram of the Hele-Shaw cell experiment.

The Hele-Shaw cell experiment was performed as shown in Fig. 3. IP6/β-TCP powder (IP6 concentration: 0, 1000, 3000, 5000, and 10000 ppm) was used as the starting powder. The powder/liquid ratio of the powder to the mixing solution was 1.0/0.95 [w/v], and the mixing was performed for 2 min. The resulting cement paste was filled into a syringe with 0.25 cm^3^ and extruded onto a glass plate, on which another glass plate was placed. At first, the glass plate was crushed with both hands until the cement paste was as flat as possible, and when it was flat, it was photographed over time using a digital camera fixed at the same observation point. Note that a beaker containing 500 cm^3^ of water was always placed on the glass slide as a weight and pressed during the waiting period for photographing. The perimeter of the glass slides was secured with Scotch tape to prevent the glass slides from shifting as the paste hardened. Pieces of each cement specimen after setting were randomly selected for microstructural observation by SEM.

**Fig. 4:**
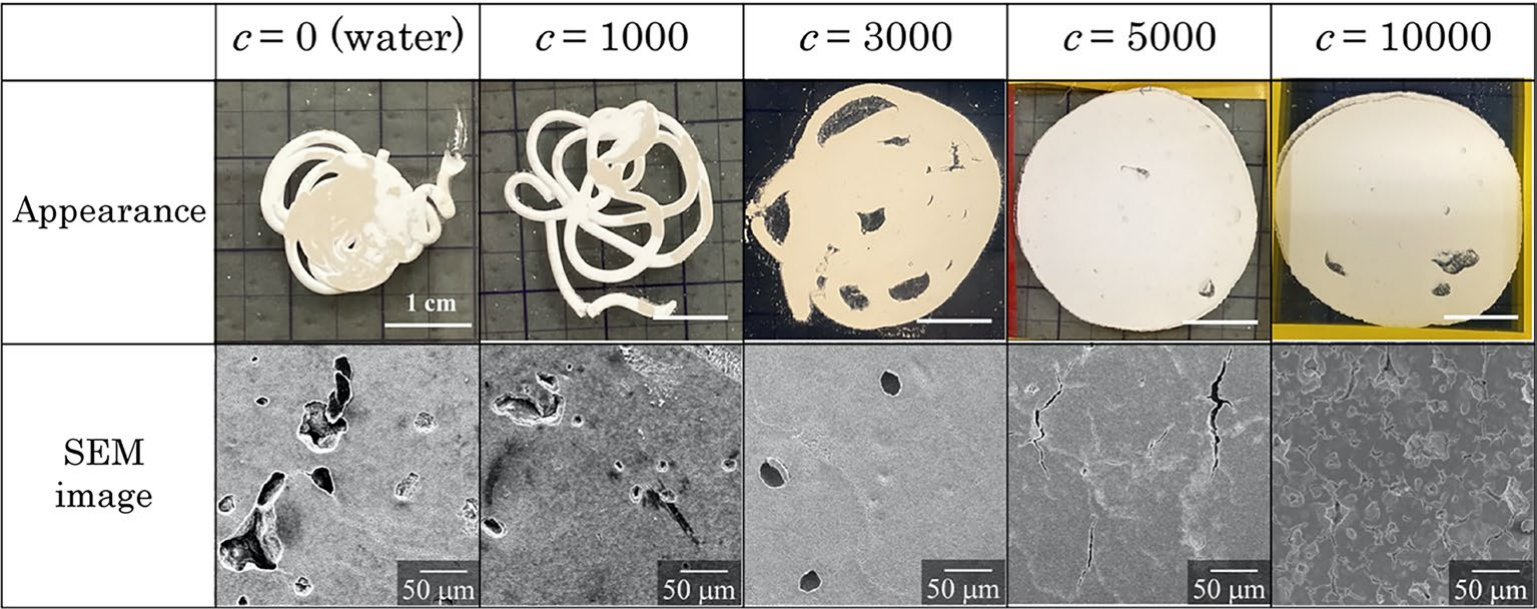
Appearance of pastes after 30 minutes (upper column) and their microstructures observed by SEM (lower one). c indicates the concentration of IP6 solution. The higher the IP6 concentration, the more well elongated the cement paste can be observed.

Figure 4 shows the appearance of pastes after 30 min and their microstructures observed by SEM. Firstly, in the appearance, when the IP6 concentrations modified to β-TCP powder were 0 and 1000 ppm, the paste ejected from the syringe did not extend and cured in a shape similar to a snake’s coil. In contrast, at the IP6 concentrations of 3000 ppm and above, the paste was observed to be extending; at 10,000 ppm, the paste was not completely set even after 30 min. These observations indicate that the paste extends well and few macro cracks form when the IP6 concentration is set appropriately. The observation of these representative SEM images also shows that the higher the IP6 concentration, the less crack formation is understood. Therefore, it can be concluded that the control of IP6 concentration is a key factor in inhibiting the formation of microcracks, which cause CPC fragmentation. This finding will be used to build a mathematical model.

### Mathematical model and numerical results

In this section, we construct a qualitative mathematical model by means of the so-called the Allen-Cahn type equation. To simplify the phenomena of the experiments, let $\:\varOmega\:=\left[-L,L\right]\times\:\left[-L,L\right]\subset\:\:{\mathbb{R}}^{2}$ be a small square domain in a two-dimensional disk, which is sliced into a circle of 3D cylindrical shapes.

We use the order parameter function which is a field variable that represents the state of the paste as it is being set. Let the order parameter function be $\:\varphi\:=\varphi\:\left(t,\:x,y\right)$ at the point $\:\left(x,y\right)\in\:\varOmega\:$ and the time $\:t$.The function $\:\varphi\:$ takes values within $\:\left[\text{0,1}\right]$ as follows: $\:\varphi\:=1$ represents a solid, $\:\varphi\:=0.5$ represents a liquid, $\:\varphi\:=0$ represents a void and $\:0<\varphi\:<1$ is an intermediate state in which the setting is not complete. Our proposed model is the following Allen-Cahn type equation with a time-dependent diffusion coefficient in spatial two dimensions.:

1 $$\:\begin{array}{c}{\varphi\:}_{t}=D\left(t\right)\varDelta\:\varphi\:+{\varepsilon\:}^{-2}f\left(\varphi\:\right),\hspace{1em}t>0,\hspace{1em}\left(x,y\right)\in\:\varOmega\:\subset\:{\mathbb{R}}^{2},\end{array}$$

2 $$\:\begin{array}{c}\varphi\:\left(0,x,y\right)={\varphi\:}_{0}\left(x,y\right),\hspace{1em}\left(x,y\right)\in\:\varOmega\:\subset\:{\mathbb{R}}^{2},\end{array}$$

3 $$\begin{array}{*{20}c} {\varphi _{x} \left( {t, - L,y} \right) = \varphi _{x} \left( {t,L,y} \right) = 0,\:\:\:\varphi _{y} \left( {t,x, - L} \right) = \varphi _{y} \left( {t,x,L} \right) = 0,\quad t > 0.} \\ \end{array}$$

Let $\:{\varphi\:}_{t}=\partial\:\varphi\:/\partial\:t$. $\:\varDelta\:$ is the Laplacian. In the numerical simulations below, the initial state is a liquid with a perturbation, that is, the initial value $\:{\varphi\:}_{0}$ in (2) is $\:0.5$ plus noise data. We see the behavior of the state, that is, how the value of $\:\varphi\:$ changes in time satisfying the governing Eq. (1) with the Neumann boundary conditions (3). The domain $\:\varOmega\:$ is a micro-square, and the boundary of it is not the “real” boundary of the 2D sliced disk but a mathematical boundary. Here, we assume the Neumann boundary conditions for the numerical studies.

We state the parameters in (1) as follows: $\:\varepsilon\:=\varepsilon\:\left(c\right)>0$ is a positive given constant depending on the concentration $\:c$ of IP6, consistent with the experimental results in the Hele-Shaw cell (see also Subsection 2.3). The parameter $\:\varepsilon\:$ is an indicator of the strength of the phase separation. (1) is the well-known Allen-Cahn type equation but it is not the usual type, since the diffusion coefficient $\:D\left(t\right)$ depends on time $\:t$, and it approximates the experimental results of the Vicat needle (see also Subsection 2.2). We simply assume that the diffusion term $\:D\left(t\right)\varDelta\:\varphi\:$ will disappear in a finite time $\:T$:

$$D(t) = \left\{\begin{array}{ll}{D}_{0} &  \left(0 \le t<T<+{\infty}\right), \\ 0 &  \left(t \ge T\right), \end{array}\right.$$

where $\:{D}_{0}\:$is a positive constant, and $\:T=T\left(c\right)$ is the time when the initial setting is completed. The time interval $\:0\le\:t<T\left(c\right)<+{\infty\:}$ corresponds to the initial setting and $\:t\ge\:T\left(c\right)$ corresponds to the recuperation stage. We assume that the diffusion is viewed as the setting. Note that the experimental results reflect that the time at which the depth of the Vicat needle reaches zero is dependent on the concentration of IP6. Although one could think of any number of functions that would better approximate the graph obtained in the experiment the point of rapid depression in the graph. We believe that small differences in the way the function is approximated will not make a qualitatively large difference in the later numerical results, so we decide to adopt $\:D\left(t\right)$ by a simpler discontinuous function.

The reaction term $\:f\left(\varphi\:\right)$ is assumed as follows:

$$\:f\left(\varphi\:\right)=f\left(\varphi\:;c\right)=\varphi\:\left(1-\varphi\:\right)\left(\varphi\:-\overline{\varphi\:}\right),\hspace{1em}0<\overline{\varphi\:}=\overline{\varphi\:}\left(c\right)<1.$$

This is a typical reaction term in the Allen-Cahn equation. Let us consider the role of $\:f\left(\varphi\:\right)$ by the ordinary differential equation $\:{\varphi\:}_{t}=f\left(\varphi\:\right)$: If $\:0<\varphi\:<\overline{\varphi\:}$, then $\:f\left(\varphi\:\right)<0$ holds and $\:\varphi\:$ decreases in time. In the same way, if $\:\overline{\varphi\:}<\varphi\:<1$, then $\:f\left(\varphi\:\right)>0$ holds and $\:\varphi\:$ increases in time. Hence, the closer $\:\overline{\varphi\:}$ is to $\:0$ (resp. 1), the more $\:\varphi\:$ goes to become $\:1$ (resp. 0). The ease of becoming solid $\:\overline{\varphi\:}$ refers to uniform spreading, and the Hele-Shaw cell experiment shows that it depends on the concentration of IP6. As the concentration of IP6 increases, it becomes more likely to become solid, which leads us to consider the following qualitative assumption, where $\:\overline{\varphi\:}\left(c\right)$ approaches $\:0$ as the concentration $\:c$ increases, namely a monotone decreasing function:

$$\:\overline{\varphi\:}\left(c\right)=\frac{0.5}{1+c/1000}.$$

The justification for the modeling is discussed below in light of the experimental results in Sect. 2. As the concentration $\:c$ of IP6 increases, the setting slows down based on the experimental results of the Vicat needle. A slower setting means longer diffusion in time. That is, $\:T\left(c\right)$ is longer and $\:\varepsilon\:\left(c\right)$ increases. In addition, as the concentration $\:c$ of IP6 increases, $\:\overline{\varphi\:}\left(c\right)$ becomes smaller and more likely to become solid. The dependence of $\:\varepsilon\:$, $\:D$, $\:T$ and $\:\overline{\varphi\:}$ on the concentration of IP6 reflects the above three experimental results.

The four parameters depend on the IP6 concentration $\:c$. The dependency can be removed under the following scale-transformation. We assume that $\:T\left(c\right)$ and $\:\varepsilon\:{\left(c\right)}^{2}$ are in a proportional relationship:

4 $$\:\begin{array}{c}T\left(c\right)\sim\:\varepsilon\:{\left(c\right)}^{2}.\end{array}$$

This assumption is natural from a viewpoint of nondimensionalization, and is based on the qualitative fact that increasing the concentration of IP6 results in a longer $\:T\left(c\right)$ and a predominance of diffusion over phase separation $\:\varepsilon\:{\left(c\right)}^{2}$. Then we use the assumption (4) and the following transformations:

$$\:\varPhi\:\left(\tau\:,\xi\:,\eta\:\right)=\varphi\:\left(t,x,y\right),\hspace{1em}d\left(\tau\:\right)=D\left(t\right),\hspace{1em}x=\sqrt{\mu\:}\xi\:,\hspace{1em}y=\sqrt{\mu\:}\eta\:,\hspace{1em}t=\lambda\:\tau\:.$$

We then have

$$\:\frac{\partial\:\varPhi\:}{\partial\:\tau\:}\left(\tau\:,\:\xi\:\right)=\frac{\:\lambda\:\:}{\mu\:}d\left(\tau\:\right){\varDelta\:}_{\varvec{\xi\:}}\varPhi\:+\frac{\lambda\:}{{\varepsilon\:}^{2}}f\left(\varPhi\:\right).$$.

Set the three new parameters $\:{d}_{0},\:{\varepsilon\:}_{0},\:{\tau\:}_{0}$ as follows:

$$\:\lambda\:=\frac{{\varepsilon\:}^{2}}{{\varepsilon\:}_{0}^{2}}=\frac{T\left(c\right)}{{\tau\:}_{0}},\:\:\:\mu\:=\frac{{D}_{0}}{{d}_{0}}\lambda\:.$$

Therefore, we obtain

$$\:\:\frac{\:\lambda\:\:}{\mu\:}d\left(\tau\:\right)=\left\{\begin{array}{cc}{d}_{0}&\:(0\le\:\tau\:<{\tau\:}_{0})\\\:0&\:(\tau\:\ge\:{\tau\:}_{0})\end{array}\:\:\right..$$.

This means that we have a nondimensional model equation for $\:\varPhi\:$, and by undoing the letters to the original ones, we obtain the model (1)–(3). The equation does not change apparently, but only $\:\overline{\varphi\:}\left(c\right)$ depends on $\:c$ in this transformation. See Table 1 for a summary of the mathematical model components, the experimental results up to this point, and the graph shapes.

**Table 1:** Comparison of typical elements of mathematical models and experiments.

Elements of mathematical model	Corresponding experiments	Comments
$\:\varphi\:=\varphi\:\left(t,\:x,y\right)$	No	The order parameter function
$\:c$	No	The concentration of IP6
$\:D\left(t\right)$	The Vicat needle (see Fig. 2)	$$D(t) = \left\{\begin{array}{ll}{D}_{0} & \left(0 \le t<T<+{\infty}\right), \\ 0 & \left(t \ge T\right), \end{array}\right.$$
$\:f\left(\varphi\:\right)$	The Hele-Shaw cell (see Fig. 3)	$f\left( \varphi \right) = f\left( {\varphi ;c} \right) = \varphi \left( {1 - \varphi } \right)\left( {\varphi - \overline{\varphi }} \right),$

**Fig. 5:**
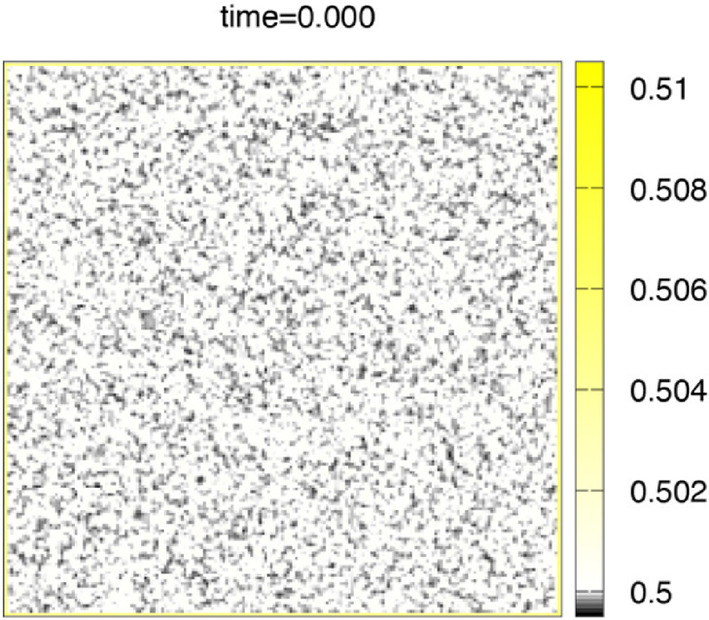
This figure indicates the initial common data at time $\:t=0$ with a random 5% noise of the space increment $\:h$ to the initial constant value $\:{\varphi\:}_{0}$.

**Fig. 6:**
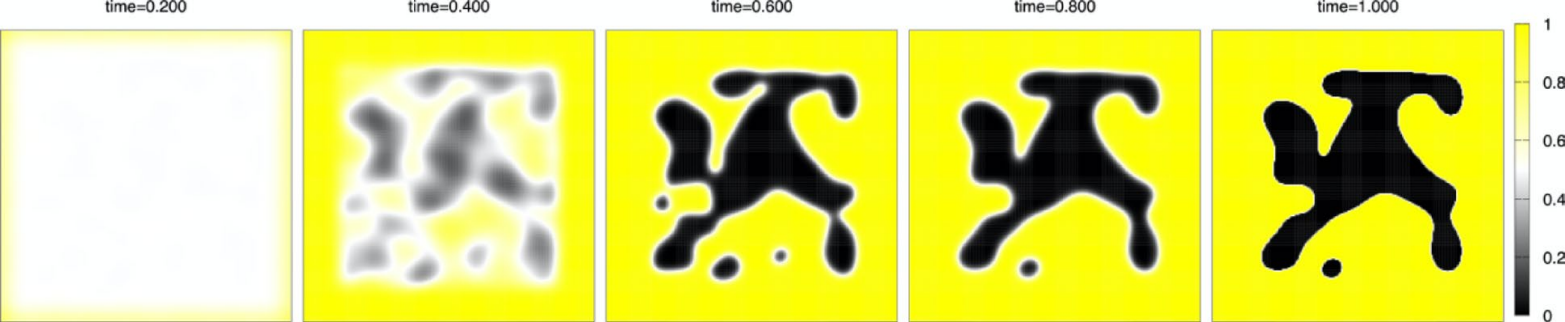
Time evolution for the case of Water $\:c=0$. From left to right: $\:t=0.2,\:0.4,\:0.6,\:0.8,\:1.0$.

**Fig. 7:**
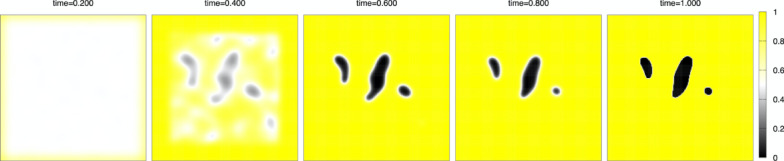
Time evolution for the case of a low amount of IP6 $\:c=0.02$. From left to right: $\:t=0.2,\:0.4,\:0.6,\:0.8,\:1.0$.

**Fig. 8:**
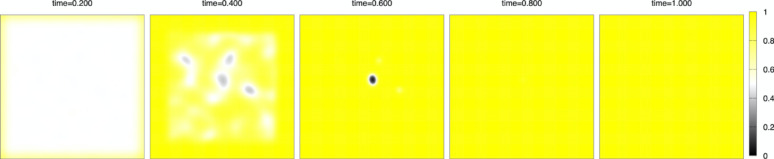
Time evolution for the case of a sufficient amount of IP6 $\:c=0.03$. From left to right: $\:t=0.2,\:0.4,\:0.6,\:0.8,\:1.0$.

Numerical simulation. We discretize the model (1)–(3) by the method of lines, which means discretization in space with keeping continuous time, and solve the ordinary differential equation (ODE) by the classical fourth-order Runge-Kutta method. The advantage of the method is that we do not need to switch from partial differential Eq. (1) with $\:D\left(t\right)>0$ to ODE (without $\:D\left(t\right)$) at the finishing time $\:T\left(c\right)$ of the initial setting. Under the fixed parameters $\:T\left(c\right)=0.8,\:\:\varepsilon\:=0.1,\:L=1,\:{D}_{0}=0.01$, and the number of grid points $\:N=200$, the space increment $\:h=2L/N$, the time increment $\:{\Delta\:}t=0.1/{N}^{2}$, numerical calculation are done in the time interval $\:[0,\:1]$ for three kinds of the concentration cases $\:c=0,\:0.02,\:0.03.\:$ The initial state is the case where setting started near the boundary of $\:{\Omega\:}$ while inside of $\:{\Omega\:}$ states liquid-like before setting. Figure 5 indicates a random $\:5\%\:$noise of $\:h$ to the initial constant value $\:{\varphi\:}_{0}$, which is the initial common data. Yellow color indicates a solid state ($\:\varphi\:=1$), black color indicates a void ($\:\varphi\:=0$), and almost white color indicates an intermediate state in which the setting is not complete ($\:0<\varphi\:<1$), especially pure white color indicates a liquid state ($\:\varphi\:=0.5$). In Figs. 6,7, 8, snapshots of the time evolution of $\:\varphi\:$ are depicted at the time $\:t=0.2,\:0.4,\:0.6,\:0.8,\:1.0$. Figure 6 indicates the water case ($\:c=0$), Fig. 7 indicates low IP6 case ($\:c=0.02$), and Fig. 8 indicates sufficient amount of IP6 case ($\:c=0.03$). In each figure, at the time 0.2, the setting makes rapid progress from boundary to inside, and at the time 0.4, the vague void region appears with mixed liquid. At the time 0.6, we can find the clear void region (black) which is surrounded by a liquid boundary (white). In the water case of Fig. 6 and the low IP6 case of Fig. 7, the void region keeps until the final time $\:t=1$. The difference between $\:t=0.8$ and $\:t=1$ is the boundary of void region. At the final time $\:t=1$, the white liquid boundary disappears, and the void black regions are surrounded by the yellow setting state. In contrast, if amount of IP6 is sufficiently large, the void region vanishes after $\:t=0.8$ as in Fig. 8. These results suggest that non-fragmentation property can be achieved if IP6 is enough, especially hard to achieve the property for pure water case.

## Discussion

The evaluation of material properties and the demonstration of the usefulness of paste-like artificial bone are important issues that need to be addressed for their practical application. This paper takes advantage of the universality of the mathematical model, the security of logic, and the provision of hypotheses to address essential questions in the setting behavior of pastes depending on materials through the construction of it and its analysis.

Although it is difficult to express the *in vitro* experiments in^5^ as mathematical equations, we conduct experiments to establish the rationale and assumptions for mathematical model construction. Although all three experiments in the present study were the result of non-equilibrium conditions in terms of chemistry, we then successfully use the results in terms of the equations and necessary assumptions to construct a simple mathematical model, as illustrated at time = 1000 in Figs. 6, 7 and 8. Our proposed mathematical model is based on the mathematically well-known Allen-Cahn type equation. The state of the paste is used as an unknown function, and the model is a simple combination of terms corresponding to the experimental results. In order to elucidate the mechanism of the influence of material properties on the setting phenomenon of paste-like artificial bone, which is a complex and multi-process, a natural modeling is performed with one simple Allen-Cahn type equation with the concentration of IP6 as an important factor. This modeling and numerical simulation successfully provide a logic to answer in biomaterial’s question.

In this paper, the time-dependent diffusion coefficient, denoted $\:D\left(t\right)$, the function $\:\overline{\varphi\:}\left(c\right)$ in the reaction term $\:f\left(\varphi\:\right)$, and assumption (4) are qualitatively introduced from the experimental results in Sect. 2. Note that $\:D\left(t\right)$ and $\:\overline{\varphi\:}\left(c\right)$ are functions that we have naturally approximated from experimental results. It is an open problem as to how to propose and validate experimental methods to support this assumption (4) the functions $\:D\left(t\right),$ $\:\overline{\varphi\:}\left(c\right)$. In addition, the numerical simulations are performed with noise set to 5%. The analysis of the sensitivity to noise is a future work.

Numerical simulation of our proposed mathematical model with a scaling transformation that reduces the term related to the concentration of IP6 yields the result that the fragmentation property shows in the absence of IP6, and the non-fragmentation property shows when IP6 is sufficiently present. The numerical results represent the setting behavior of the paste, which is difficult to observe experimentally. This numerical result corresponds to the small square region in the final result obtained in the Hele-Shaw cell experiment.

The coarseness and density that inevitably occur when the paste is injected is one of the factors that causes voids to appear as the paste sets. Our mathematical model and the qualitative results shown by the simulations can answer the question of why IP6 is involved in the non-fragmentation property. Even if coarseness and density of the paste are created in the initial state, when IP6 is sufficiently contained, the result of numerical simulation and the experimental results of the Vicat needle show that it exhibits the non-fragmentation property by trying to be uniform by allowing time for diffusion for setting. Considering the results of the coloring experiment to observe particle dispersion and numerical simulation, the flowability is strongly exhibited, which, together with the above-mentioned sufficient security of the setting time, results in the non-fragmentation property in the case of sufficient IP6. These considerations are supported by the universality of the mathematical model, which guarantees the conclusions obtained from the three experiments carried out to build the mathematical model. The above discussion is the reason for the non-fragmentation property in the case involving IP6.

Our proposed mathematical model and its numerical results guarantee the logic of the considerations obtained from the experiments in this paper. From a biomaterial perspective, the introduction of a different mathematical perspective to the fundamental question of material properties essential for the medical contribution of paste-like artificial bones provides a new understanding of the setting behavior of pastes, and an area that is difficult to observe experimentally. Needless to say, this understanding will play an important role in the evaluation of IP6 and other novel material properties. The conclusions reached in this paper are difficult to reach by previous experimental results alone and can be obtained by integrating the results of experiments for mathematical model construction, mathematical models, and their numerical results through integration with mathematics. We believe that our conclusions give a breakthrough in fundamental research on the material properties of paste-like artificial bones and should serve as a guideline for the creation of new materials.

It is not actual to decompose the experiment into elementary processes and construct a mathematical model from the bottom up, considering the complexity of the phenomena in the experiment. Therefore, we attempted top-down qualitative modeling. It is extremely important to clarify the quantitative relationship. We will leave the construction of a quantitative model as a future work.

On the other hand, from a mathematical point of view, our model is based on the well-known Allen-Cahn type equation with time-dependent diffusion coefficients. This research creates a new application of the Allen-Cahn type equation. In addition, the existence of time-dependent diffusion coefficients makes them difficult to handle in numerical simulations. In addition to the Allen-Cahn equation, the Cahn-Hilliard equation also describes the phase separation phenomenon. The question of whether the influence of the fourth-order derivatives makes the expression of the paste setting behavior more precise, and what difference it makes, is a subject for future work. The mathematical model proposed in this paper creates an independent new problem in the search for more accurate and tractable numerical solutions from the point of view of numerical analysis.

## Data Availability

Datasets of experimental results and mathematical models will be provided by the corresponding authors upon reasonable request.

## Code availability

Code is provided via GitHub. It is available at https://github.com/math-exp/Calcium-phosphate-cement for review. SY maintains it in an established DOI (https://doi.org/10.5281/zenodo.15589137).

## References

[1] Tsuru, K., Sugiura, Y. & Ishikawa, K. in Nanobioceramics for healthcare applications. 151–186 (eds Thian, E. S., Huang, J. & Aizawa, M.) (World Scientific Publishing Europe Ltd, 2017).

[2] Konishi, T. et al. Injectable chelate-setting hydroxyapatite cement prepared by using Chitosan solution: fabrication, material properties, biocompatibility, and osteoconductivity. *J. Biomater. Appl.* **31**, 1319–1327. 10.1177/0885328217704060 (2017). 28517977 10.1177/0885328217704060

[3] Kamaya, Y. et al. Development of fully-resorption replacement paste-like organic/inorganic artificial bones compatible with bone remodeling cycles. *Biomaterials Biosystems*. **17**, 100107. 10.1016/j.bbiosy.2025.100107 (2025). 39963254 10.1016/j.bbiosy.2025.100107 PMC11830353

[4] Honda, M. et al. Bactericidal and bioresorbable calcium phosphate cements fabricated by silver-containing tricalcium phosphate microspheres. *Int. J. Mol. Sci.* **21** (11), 3745 (2020). 32466460 10.3390/ijms21113745 PMC7312163

[5] Nagata, K. et al. Evaluation of resistance to fragmentation of injectable calcium-phosphate cement paste using X-ray microcomputed tomography. *J. Ceram. Soc. Jpn*. **125**, 1–6 (2017).

[6] Allen, S. & Cahn, J. W. A microscopic theory for antiphase boundary motion and its application to antiphase domain coarsening. *Acta Metall.* **27**, 1084–1095 (1997).

[7] Okumura, M. A stable and structure-preserving scheme for a non-local Allen-Cahn equation. *Jpn J. Ind. Appl. Math.* **35**, 1245–1281 (2018).

[8] Konishi, T. et al. Biodegradable β-tricalcium phosphate cement with anti-washout property based on chelate-setting mechanism of inositol phosphate. *J. Mater. Sci. : Mater. Med.* **24**, 1383–1394 (2013). 23471502 10.1007/s10856-013-4903-8

